# A systematic PCR record‐based re‐call of HCV‐RNA‐positive people enables re‐linkage to care and HCV elimination in Austria — The ELIMINATE project

**DOI:** 10.1111/liv.16076

**Published:** 2024-10-01

**Authors:** Lorenz Balcar, Michael Schwarz, Livia Dorn, Mathias Jachs, Lukas Hartl, Lukas Weseslindtner, Nikolaus Pfisterer, Barbara Hennlich, Annika Stückler, Robert Strassl, Astrid Voill‐Glaninger, Wolfgang Hübl, Martin Willheim, Karin Köhrer, Sonja Jansen‐Skoupy, Sabine Tomez, Walter Krugluger, Christian Madl, Lukas Burghart, Lukas Antonitsch, Gerhard Weidinger, Florian Riedl, Hermann Laferl, Julian Hind, Christoph Wenisch, Christian Sebesta, Julia Wachter‐Welzl, Paul Watzl, Magdalena Neuhauser, David Chromy, Mattias Mandorfer, Daniela Schmid, Michael Gschwantler, Thomas Reiberger, Andreas Maieron, David J.M. Bauer, Caroline Schwarz

**Affiliations:** ^1^ Division of Gastroenterology and Hepatology, Department of Medicine III Medical University of Vienna Vienna Austria; ^2^ Department of Internal Medicine IV Klinik Ottakring Vienna Austria; ^3^ Internal Medicine II, Gastroenterology and Hepatology and Rheumatology, Karl Landsteiner University of Health Sciences University Hospital of St. Pölten St. Pölten Austria; ^4^ Center for Virology Medical University of Vienna Vienna Austria; ^5^ Department of Internal Medicine IV Klinik Landstraße Vienna Austria; ^6^ Clinical Institute for Laboratory Medicine Medical University of Vienna Vienna Austria; ^7^ Central Laboratory and Blood Bank Klinik Landstraße Vienna Austria; ^8^ Central Laboratory Klinik Ottakring Vienna Austria; ^9^ Clinical Institute of Laboratory Medicine University Clinic St. Pölten St. Pölten Austria; ^10^ Institute of Medical‐Chemical and Molecular biological Laboratory Diagnostics with Blood Depot Landesklinikum Wiener Neustadt Wiener Neustadt Austria; ^11^ Institute of Laboratory Diagnostics Klinik Favoriten Vienna Austria; ^12^ Institute of Laboratory Medicine with Blood Depot Klinik Donaustadt Vienna Austria; ^13^ Institute of Laboratory Medicine and Blood Depot Klinik Floridsdorf Vienna Austria; ^14^ Sigmund Freud University Vienna Austria; ^15^ Department of Internal Medicine, Gastroenterology and Hepatology Landesklinikum Wiener Neustadt Wiener Neustadt Austria; ^16^ Department of Internal Medicine IV Klinik Favoriten Vienna Austria; ^17^ Department of Internal Medicine and Gastroenterology Klinik Floridsdorf Vienna Austria; ^18^ Department of Internal Medicine II Klinik Donaustadt Vienna Austria; ^19^ Department of Internal Medicine II Landesklinikum Mistelbach Mistelbach Austria; ^20^ Department of Internal Medicine II Barmherzige Schwestern Hospital Vienna Austria; ^21^ Austrian Agency for Health and Food Safety (AGES) Vienna Austria

**Keywords:** DAA, elimination, hepatitis C, WHO

## Abstract

**Background and Aims:**

Identification of people living with hepatitis C virus (HCV) via readily available laboratory records could be a key strategy for macro‐elimination, aligning with the WHO elimination goal. Therefore, the ELIMINATE(ELIMINation of HCV in AusTria East) project aimed to systematically re‐link people with a ‘last‐positive’ HCV‐RNA PCR record to care.

**Methods:**

In 10 major liver centres in Eastern Austria, a systematic readout of ‘last‐positive’ HCV‐RNA PCR test records obtained between 2008 and 2020 were conducted and linked to available patient contact data. Between 2020 and 2023, individuals were contacted first by phone, then by letter, to inform them about the availability of effective direct‐acting antiviral (DAA) treatment and invite them for pre‐treatment evaluation.

**Results:**

The overall cohort of last‐positive HCV+ individuals included 5695 subjects (62.5% males, mean age 57.3 ± 17.3 years); of note, 1931 (34%) of them had died and 759 (13%) individuals had no valid contact information. Of the remaining 3005 individuals, 1171 (40.0%) had already achieved sustained virological response (SVR) at the time of re‐call. We successfully reached 617 (20.5%), of whom 417 (67.6%) attended their pre‐treatment visit, and 397 (64.3%) commenced DAA‐therapy. HCV cure has been confirmed in 326 individuals, corresponding to an SVR rate of 82.1%.

**Conclusion:**

The ELIMINATE project identified 5695 people living with HCV who were ‘lost to care’ despite documented HCV viraemia. While invalid contact data were an evident barrier to HCV elimination, premature deaths among the cohort underscored the severity of untreated HCV. The implementation of a systematic HCV‐RNA PCR recorded‐based re‐call workflow represents an effective strategy supporting the WHO goal of HCV elimination.

## Graphical abstract

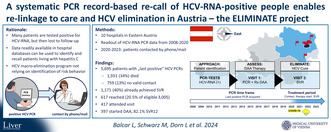

**Abbreviations**
APRI: aspartate aminotransferase to platelet ratio‐index scorer
CAP: continuous attenuation parameter
DAA: direct‐acting antiviral agents
FIB‐4: fibrosis‐4 score
HBV: hepatitis B virus
HCV: chronic hepatitis C virus
HIV: human immunodeficiency virus
LSM: liver stiffness measurement
MSM: men who have sex with men
NIT: non‐invasive test
PWID: people who inject drugs
RNA: ribonucleic acid
SVR: sustained virological response
WHO: World Health Organization

**Lay summary**

Chronic hepatitis C, a viral infection causing liver damage, cirrhosis and liver cancer, affects millions globally. With the development of highly effective and curative antiviral drugs over the last decade the World Health Organization has set an ambitious target to eliminate hepatitis C as a global health threat by 2030. Achieving this goal hinges on the success of elimination programs that identify individuals and link them to care. In this study, we present a large‐scale laboratory record‐based project in major liver centres in Eastern Austria (ELIMINATE), within which we systematically re‐called individuals infected with the hepatitis C virus (HCV) who had previously fallen out of care. We successfully initiated antiviral treatment in a considerable number of these individuals and cured more than 320 individuals from hepatitis C. Projects like this crucially contribute to realising the goal of HCV elimination.

## 1 INTRODUCTION

Worldwide, chronic infection with the hepatitis C virus (HCV) affects 56.8 million people and causes 250 000 liver‐related deaths per year.^1^ As a result of HCV‐associated chronic hepatic inflammation, 5%–10% of individuals with chronic hepatitis C develop liver cirrhosis over the course of 20 years; subsequently, 2%–3% face an annual risk for hepatic decompensation or hepatocellular carcinoma.^2^, ^3^

The introduction of direct‐acting antiviral (DAA) agents marked a significant change in the treatment of hepatitis C. Unlike previous interferon‐based therapies, which were characterised by lengthy treatment durations, poor tolerability and relatively low cure rates of 50%–60%,^4^ DAAs have revolutionised treatment by successfully clearing the virus in virtually all individuals and genotypes at a favourable tolerability profile due to their targeted action on proteins essential to the viral replication circle.^5^, ^6^

In 2016, with the advent of effective and finite treatments for what was previously a chronic disease, the World Health Organization (WHO) announced their ambitious goal to eliminate viral hepatitis, including HCV as a global public health threat by 2030.^7^ To achieve this goal, pivotal pillars of the elimination strategy were increasing the rates of HCV diagnosis (from 5% to 90% globally) and treatment (from <1% to 80% of HCV‐infected individuals). These efforts aim to achieve a 90% reduction in HCV incidence and 65% reduction in globally HCV‐related mortality.^7^ The successful implementation of the WHO's plan could prevent an estimated 15.1 million HCV infections and 1.5 million HCV‐related deaths by 2030.^8^

The journey to HCV‐elimination is reflected by the ‘cascade of care’, encompassing the following key steps: estimating prevalence in a defined cohort or region of interest, diagnosing HCV infection (i.e. HCV‐RNA/viraemia), initiating treatment and confirmation of proving HCV clearance by undetectable blood stream HCV‐RNA (usually 12 weeks) after end of treatment (i.e. sustained virological response, SVR12).^1^, ^9^, ^10^ Estimates for people living with HCV in Austria range from 25 000 to 80 000, corresponding to a prevalence of 0.2%–0.9% in the overall population.^1^ The most common route of transmission in Austria as well as Europe is ongoing injecting drug use and penetrative unprotected sexual intercourse, especially among men who have sex with men (MSM).^11^, ^12^ In the past, several micro‐elimination programs targeting high‐risk groups such as people who inject drugs (PWIDs),^13^, ^14^, ^15^ MSM,^16^ homeless people,^17^ and prison inmates,^18^ successfully identifying and treating individuals within these cohorts, have been launched in Austria. These micro‐elimination programs are effective tools on the road to reaching the WHO goals, operating on the principle that micro‐elimination will ultimately and cumulatively result in macro‐elimination.^19^, ^20^, ^21^, ^22^, ^23^, ^24^

These ‘lost to care’ individuals often underwent anti‐HCV antibody testing in the past, perhaps as a routine measure prior to surgical interventions, yet treatment was not initiated or unavailable at the time.^26^, ^27^, ^28^ Reconnecting with these individuals is key to increasing treatment rates among those living with HCV and to reduce future HCV transmission. Countries like Spain^29^ and Egypt^30^ have implemented national elimination plans that include comprehensive interventions, including general population HCV screening. This approach facilitates the identification of individuals beyond the scope of micro‐elimination programs. In contrast, Austria, despite its numerous successful micro‐elimination efforts, lacks a national elimination plan, and hence, identification of such individuals ‘submerged’ within the general populace has historically been a challenge. With the Austrian healthcare system fully covering DAA treatment costs for those with national universal health insurance, which is available to all people with registered employment or unemployment status in Austria, and temporary coverage available through charitable organisations,^17^ the identification of individuals living with HCV and their linkage to care now represents the key barrier on Austria's road to HCV elimination. While local micro‐elimination programs targeting populations specifically vulnerable to HCV infection have been effective, the gap between the number of people reached through these tailored interventions and the estimated national HCV prevalence in Austria indicates that a relevant number of people living with HCV are yet to be discovered.

We present the final report of our macro‐elimination program ‘ELIMINATE’ (‘ELIMINation AusTria East’),^31^ focusing on identification and treatment of an HCV viraemic population lost to HCV care or unaware of their HCV‐RNA+ status.

## 2 METHODS

### 2.1 Aims and outcomes

**FIGURE 1:**
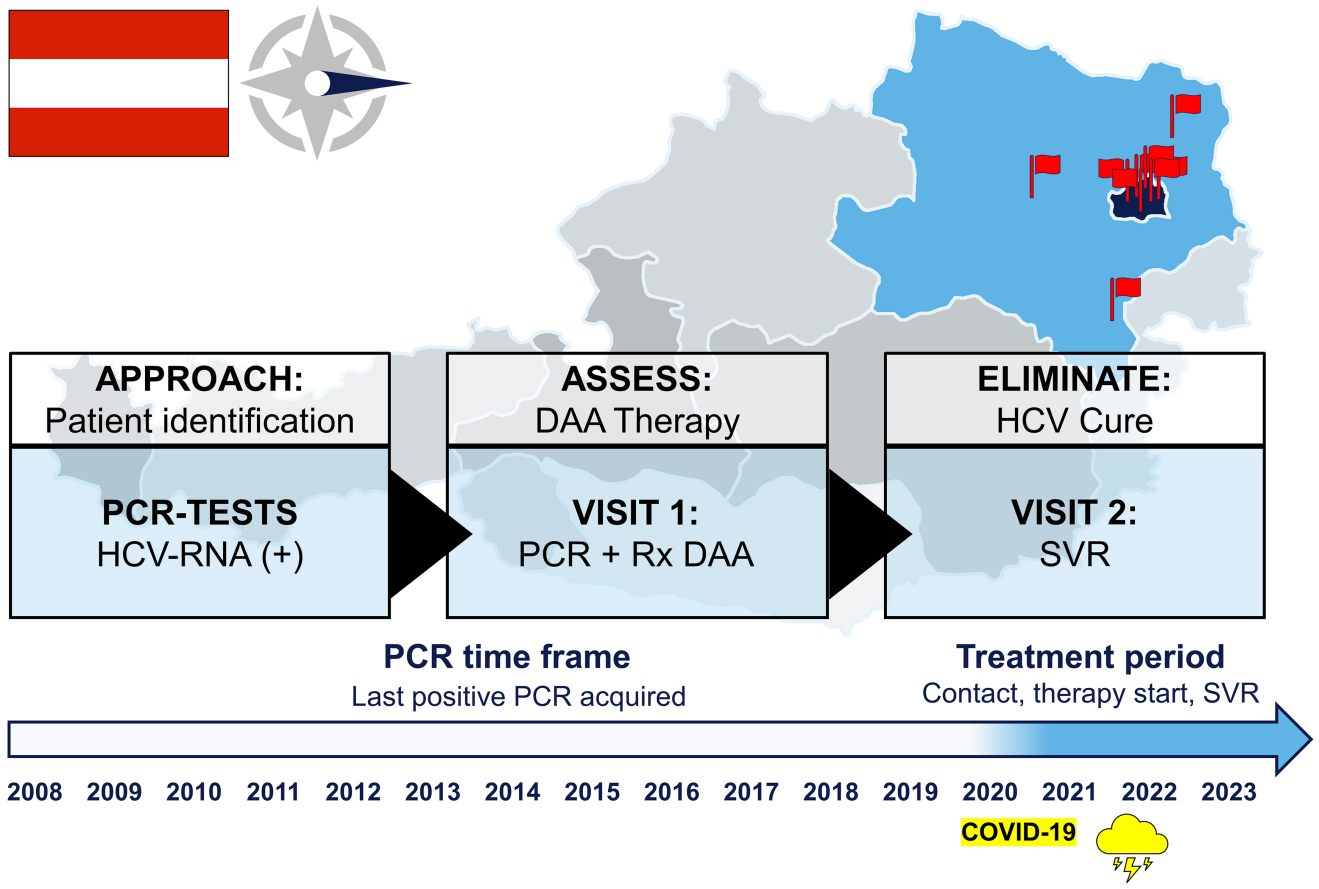
Overview of the macro‐elimination ELIMINATE program in Eastern Austria. Ten tertiary care centres in Eastern Austria collaborated on a macro‐elimination strategy targeting Hepatitis C. We re‐called individuals with a documented ‘last‐positive’ HCV RNA PCR from 2008 to 2020. Systematic outreach was conducted via telephone or mailed letter to re‐establish linkage to care. During the initial visit at the respective centres' outpatient clinic, we reassessed each patient's current HCV status and liver disease severity. Confirmed cases of HCV viraemia led to the immediate offer and, upon acceptance, start of direct acting antiviral (DAA) therapy. A follow‐up visit confirmed sustained virological response (SVR) through PCR re‐evaluation. DAA, direct acting antiviral agents; HCV, hepatitis C virus; Rx, prescription; SVR, sustained virological response.

The objective of this study was to identify individuals with a ‘last‐positive’ HCV‐RNA PCR recorded between 2008 and 2020 at the reference virology laboratories in Eastern Austria, and to investigate the effectiveness of using a phone/letter‐based re‐call strategy by reporting the numbers/rates along the typical cascade of care for individuals infected by HCV (Figure 1).

All individuals identified via a positive HCV‐RNA PCR were included in the overall cohort and actively re‐called either by phone or mailed letters, during the study period between 2020 and 2023. Generally, eligible individuals were attempted to be contacted via phone call at least twice throughout a time period of 7 days; and if unsuccessful, a letter was mailed to the individuals stating that because of a previously positive hepatitis C test, a follow‐up appointment (at the respective treatment centre) was recommended for a medical check‐up and potential initiation of highly effective therapies. To enhance linkage to care, the liver clinic of the Vienna General Hospital had established a dedicated HCV hotline^32^, ^33^ operated by dedicated healthcare professionals at each centre (nurses and medical doctors). It allowed individuals to directly contact healthcare professionals and schedule appointments for diagnostic evaluations and/or the initiation of DAA therapy. With this, individuals living with HCV regardless of their risk behaviour, who previously did not receive treatment (lost to follow‐up, non‐eligible) or who were unaware of their chronic infection were identified and invited to the respective HCV treatment centre for treatment consideration, and ultimately, to start treatment.

### 2.2 Study design and definitions

In Austria, DAA therapy is covered by universal health insurance, but only certified HCV treatment centres may issue a DAA prescription. Hence, all main HCV treatment centres in Eastern Austria were invited to join this study. Ultimately, 10 centres from Vienna (Vienna General Hospital/Medical University, Vienna, Klinik Ottakring, Klinik Landstraße, Klinik Donaustadt, Klinik Floridsdorf, Klinik Favoriten, Barmherzige Schwestern Wien) and Lower Austria (Landesklinikum Wiener Neustadt, Landesklinikum Mistelbach, Universitätsklinikum St. Pölten) participated. Data were acquired through automated systematic retrieval of laboratory data in each centre.

We defined ‘chronic hepatitis C’ as a positive result in the HCV‐RNA PCR test, regardless of the availability of an anti‐HCV serology result. SVR was defined as a negative HCV‐RNA PCR following a previously positive HCV‐RNA PCR test. Contact details were obtained by the local patients' medical records. Individuals who were successfully contacted (i.e. reached by phone or letter, and appointment were scheduled or declined) and had not achieved SVR prior to the re‐call were invited to the respective HCV treatment centre offered comprehensive blood test (including liver disease parameters and virological assessment for HCV, human immunodeficiency virus [HIV], hepatitis B virus [HBV] and liver stiffness measurement [FibroScan®, Echosens, France]). Furthermore, individuals received counselling regarding HCV transmission and treatment options. Clinical parameters (e.g. alcohol use, ongoing injecting drug use) were assessed by the attending physician at the re‐call visit. Alcohol use was defined as several drinking days per week (three or more) with more than 14 g of alcohol per day. Ongoing injecting drug use was defined as at least one intravenous recreational drug application in the preceding 6 months.

Advanced chronic liver disease was defined based on non‐invasive test (NIT) cut‐offs either by a liver stiffness measurement of ≥15 kPa,^34^, ^35^ an AST to platelet ratio‐index (APRI)‐score of >2^36^ or a Fibrosis‐4 (FIB‐4)‐score of ≥2.67.^37^

Individuals, who were still viraemic, were offered antiviral treatment with DAAs. The choice of DAA was tailored depending on the individual comorbidities as well as on national re‐imbursement policies.

### 2.3 Statistical analysis

Statistical analyses were performed using R version 4.3.1+ (R Core Team, R Foundation for Statistical Computing, Vienna, Austria). The cascade of care includes numbers of deaths, prior‐SVRs, invalid contact information, individuals who were reached, those who attended their first visit, started treatment, achieved SVR or declined treatment. We segmented the study into three distinct periods as data were available at these specific timepoints: baseline/study inclusion at the time of ‘last‐positive’ HCV‐RNA PCR; therapy start at DAA initiation; and SVR at either four (SVR 4) or 12 weeks after end of treatment (SVR12).

At these timepoints, continuous variables were reported as mean ± SD (for those following a Gaussian distribution) and as median and interquartile range (IQR; for variables following a non‐Gaussian distribution). The number of data points available for each variable was also reported. The presence of a Gaussian distribution was determined using the Kolmogorov–Smirnov test. Categorical variables were reported as absolute (*n*) and relative (%) proportion of individuals with/without a certain characteristic. Student's *t*‐test was used for group comparisons of normally distributed variables and Mann–Whitney *U*‐test for non‐normally distributed variables, respectively. Group comparisons of categorical variables were performed using either Pearson's chi‐squared (*χ* ^2^) or Fisher's exact test, as appropriate. The level of significance was set at a two‐sided *p*‐value <0.05.

To obtain definitive data on mortality, we consulted the nationwide death registry (data cut‐off: 9 October 2023) including the ICD codes of primary cause of death. Liver‐related deaths were defined as those resulting from liver cirrhosis or its associated complications or hepatocellular carcinoma. Furthermore, we stratified according to psychiatric morbidity‐related death and time of death (pre‐ vs. COVID‐period; cut‐off: 15 March 2020). To compare survival according to SVR status, outcome data started with the date of the last HCV‐RNA PCR test until the date of death or last follow‐up. Survival was compared using the log‐rank test.

### 2.4 Ethics

Ethical approval for data collection in this study was granted by the respective ethics committees of the Medical University of Vienna (EK1968/2018), the ethics committee of the city of Vienna (EK19‐175‐VK), and the ethics committee for the state of Lower Austria (EKGS1‐EK‐4/624‐2019), as well as the ethics committee of Barmherzige Schwestern Österreich.

## 3 RESULTS

### 3.1 Cascade of care

**FIGURE 2:**
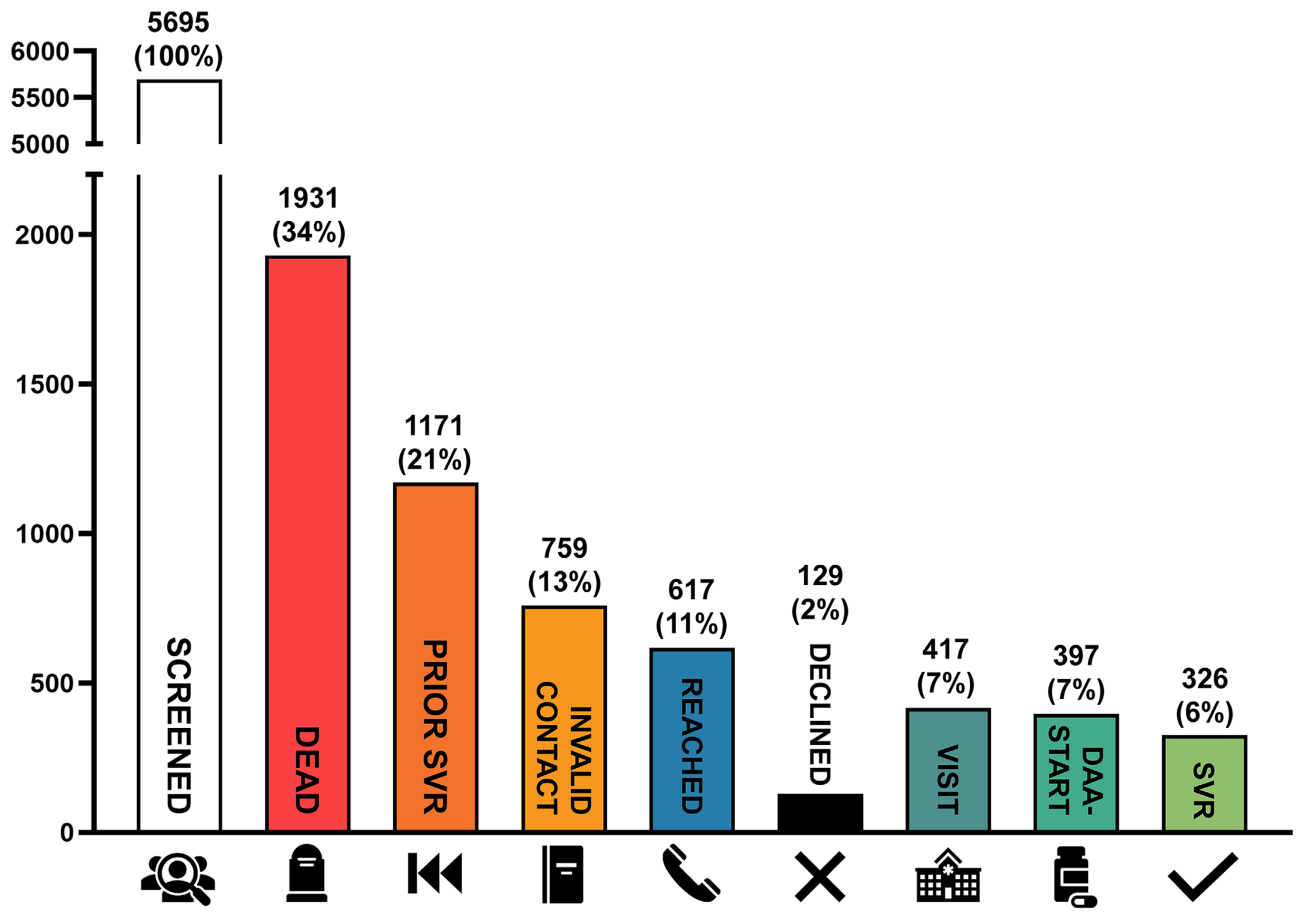
Patient care cascade overview. In total, we identified more than 5695 ‘last‐positive’ PCRs records across participating hospital databases. Valid contact information was available for 3005 patients, of whom one third had already achieved SVR12 before. We successfully contacted 617 patients, 417 of whom attended their first visit at the respective outpatient clinics. DAA‐therapy was initiated in 397 patients, with 326 achieving confirmed SVR. Alarmingly, a third of the overall cohort was found deceased between the last HCV‐RNA PCR and re‐call through our project. DAA, direct acting antiviral agents; SVR, sustained virological response.

Overall, 5695 individuals with potential persisting HCV viraemia were identified according to HCV‐RNA positivity upon their last available PCR test (‘last‐positive’) and included in the study cohort (Figure 2). At the time of re‐call, 1931 (34%) of them had passed away, and 759 (13%) had no valid contact information (phone number and/or postal address). Finally, 3005 individuals were actively re‐called, of whom 1171 (21%) reported prior successful antiviral therapy. We successfully contacted 617 (20.5% of the 3005 recalled individuals) either by phone or mailed letter, offering them a visit at the respective clinics' outpatient unit. Of these, 417 (67.6% of 617) individuals attended the offered clinical visit for reassessment of their HCV infection status. Subsequently, DAA therapy was initiated in 397 (64.3% of 617) individuals. SVR was documented in 326 individuals, representing 82.1% of those who initiated treatment.

### 3.2 Study inclusion

The overall cohort of individuals with ‘last‐positive’ HCV‐RNA PCR, as recorded at any of the 10 participating hospitals, consisted of 3559 (62.5%) males and 2136 (37.5%) females (Table 1). The mean age was 57.3 ± 17.3 years. HCV‐genotype was available in 42% of individuals, with genotype 1 (*n* = 1569, 65.6%) and 3 (*n* = 610, 25.5%) being the most prevalent. Median HCV‐RNA PCR was 690 000 (IQR: 134000‐2 510 000) U/mL.

**TABLE 1:** Baseline characteristics of patients at inclusion, corresponding to ‘last‐positive’ HCV PCR test.

	Available data, *n* (%)	Overall (*n* = 5695)
Patient characteristics		
Sex, *n* (%)		
Female	5695 (100)	2136 (37.5)
Male		3559 (62.5)
Age, years, mean (SD)		57.3 (17.3)
HCV‐genotype, *n* (%)		
1	2392 (42)	1569 (65.6)
2		78 (3.3)
3		610 (25.5)
4		133 (5.6)
5		1 (0)
6		1 (0)
Suspected main HCV transmission route, *n* (%)		
i.v. drug use	1363 (23.9)	1074 (18.9)
Sexual transmission		87 (1.5)
Blood transfusion transmission		137 (2.4)
Other transmission		65 (1.1)
Unknown transmission		4332 (76.1)
HCV‐RNA, median (IQR)	4656 (82)	690 000 (134 000–2 510 000)
LSM, median (IQR)	377 (7)	7.1 (5.5,11)
LSM ≥ 10 kPa, *n* (%)		105 (27.9)
LSM ≥ 15 kPa, *n* (%)		69 (18.3)
LSM ≥ 25 kPa, *n* (%)		31 (8.2)
APRI Score, median (IQR)		0.51 (0.3–1.08)
<0.5, *n* (%)	3660 (64)	1819 (49.7)
<1.0, *n* (%)		2675 (73.1)
>1.5, *n* (%)		663 (18.1)
>2.0, *n* (%)		494 (13.5)
FIB‐4 Score, median (IQR)		1.81 (1.11–3.54)
<1.3, *n* (%)	3616 (63)	1194 (33.0)
1.3–2.67, *n* (%)		1203 (33.3)
>2.67, *n* (%)		1219 (33.7)
Haemoglobin (g/dL), mean (SD)	2575 (45)	13.2 (2.26)
Platelets (G/L), median (IQR)	3801 (67)	205 (151–259)
<150 G/L, *n* (%)		922 (24.3)
WBC (G/L), median (IQR)	2574 (45)	6.87 (5.40–9.01)
INR, mean (SD)	992 (17)	1.2 (0.5)
Bilirubin (mg/dL), median (IQR)	2526 (44)	0.63 (0.43–1.00)
Albumin (mg/dL), mean (SD)	2481 (44)	39.4 (7.2)
AST (IU/L), median (IQR)	3698 (65)	43 (30–76)
ALT (IU/L), median (IQR)	3767 (66)	46 (27–82)
>ULN, *n* (%)		1995 (53.0)
AP (IU/L), median (IQR)	2548 (45)	83 (64–113)
gGT (IU/L), median (IQR)	3611 (63)	55 (28–119)
>ULN, *n* (%)		1886 (52.2)
CRP (mg/dL), median (IQR)	2115 (37)	0.3 (0.1–1.4)

Abbreviations: ALT, alanine aminotransferase; AP, alkaline phosphatase; APRI, AST to platelet ratio‐index; AST, aspartate aminotransferase; CAP, continuous attenuation parameter; CRP, C‐reactive protein; gGT, gammaglutamyl transferase; HCV, hepatitis C virus; INR, international normalised ratio; IQR, interquartile range; LSM, liver stiffness measurement; *n*, number; SD, standard deviation; ULN, upper limit of normal; WBC, white‐cell blood count.

Injecting drug use was the most common route of transmission (*n* = 1074, 78.8% of recorded routes). Transient elastography was available for 7% of individuals, with 69 of 377 individuals (18.3%) exhibiting elevated liver stiffness measurements (LSM ≥15 kPa) at baseline.

Using the APRI and the FIB‐4‐score cut‐offs for ruling‐out significant fibrosis (i.e. <1 and <1.3), 2675 of 3660 (73.1%) and 1194 of 3616 (33.0%) had no evidence of significant fibrosis.

Conversely, for ruling‐in significant fibrosis (i.e. >2 and >2.67), 494 of 3660 (13.5%) and 1219 of 3616 (33.7%) displayed signs of significant fibrosis. Overall, in 1264 of 3692 individuals (34.2%) one of these available NIT cut‐offs was positive for significant fibrosis. Other laboratory parameters are presented in Table 1.

### 3.3 DAA‐therapy initiation

In total, 397 (64.3% of 617 successfully contacted individuals; Table 2) people with persisting HCV viraemia commenced DAA therapy. The median delay from ‘last‐positive’ HCR‐RNA PCR to initiation of DAA treatment at one of the collaborating centres was 59.8 (IQR: 6.5–95.7) months. Most individuals were treated with sofosbuvir/velpatasvir (*n* = 299, 75.3%), followed by glecaprevir/pibrentasvir (*n* = 96, 24.2%) and sofosbuvir/velpatasvir/voxilaprevir (*n* = 2, 0.5%). Most prevalent HCV genotypes among the treated population were genotypes 1 (*n* = 202, 66.7%) and 3 (*n* = 75, 24.8%).

**TABLE 2:** Patient characteristics at DAA initiation.

	Available data, *n* (%)	Therapy starts (*n* = 397)
*Patient characteristics*		
Time from last (+)PCR to DAA treatment, months, median (IQR)	397 (100)	59.8 (6.5, 95.7)
Sex, *n* (%)		
Female	397 (100)	247 (62.2)
Male		150 (37.8)
Age, years, mean (SD)	397 (100)	50.4 (13.6)
Type of DAA, *n* (%)		
Sofosbuvir/Velpatasvir	397 (100)	299 (75.3)
Glecaprevir/Pibrentasvir		96 (24.2)
Sof./Vel./Voxilaprevir		2 (0.5)
HCV‐genotype, *n* (%)		
1	303 (76)	202 (66.7)
2		11 (3.6)
3		75 (24.8)
4		15 (5.0)
HCV‐RNA, median (IQR)	345 (87)	757 000 (244 000–2 830 000)
HBsAg+/HBV DNA+, *n* (%)	328 (83)	6 (1.8)/0 (0)
Anti‐HBs+, *n* (%)	327 (82)	72 (22.0)
Anti‐HBc+, *n* (%)	328 (83)	94 (28.7)
Anti‐HDV+/HDV RNA+, *n* (%)	327 (82)	2 (0.6)/0 (0)
HIV+, *n* (%)	327 (82)	51 (15.6)
Significant alcohol intake, *n* (%)	280 (71)	118 (42.1)
(History of) smoking, *n* (%)	278 (70)	142 (51.1)
(History of) i.v. drug use, *n* (%)	280 (71)	126 (45.0)
Psychiatric comorbidities, *n* (%)	274 (69)	60 (21.9)
Opioid agonist therapy, *n* (%)	272 (69)	64 (23.5)
Body mass index (kg/m^2^), median (SD)		25.3 (4.9)
<17.5	234 (59)	3 (1.3%)
17.5–25		124 (53.0%)
>25		107 (45.7%)
LSM, median (IQR)	238 (60)	6.9 (5.4–10.1)
LSM ≥ 10 kPa, *n* (%)		61 (25.6)
LSM ≥ 15 kPa, *n* (%)		39 (16.4)
LSM ≥ 25 kPa, *n* (%)		23 (9.7)
CAP, median (IQR)		226 (193–276)
CAP ≥248 dB/m, *n* (%)		80 (33.6)
CAP >275 dB/m, *n* (%)		50 (25.1)
APRI Score, median (IQR)		0.47 (0.3–1.01)
<0.5, *n* (%)	275 (69)	148 (53.8)
<1.0, *n* (%)		204 (74.2)
>1.5, *n* (%)		47 (17.1)
>2.0, *n* (%)		31 (11.3)
FIB‐4 Score, median (IQR)		1.4 (0.92–2.5)
<1.3, *n* (%)	275 (69)	122 (44.4)
1.3–2.67, *n* (%)		91 (33.1)
>2.67, *n* (%)		62 (22.5)
Haemoglobin (g/dL), mean (SD)	249 (63)	13.8 (2.1)
Platelets (G/L), median (IQR)	289 (73)	215 (163–261)
WBC (G/L), median (IQR)	249 (63)	6.6 (5.5–8.4)
INR, mean (SD)	214 (54)	1.1 (0.2)
Bilirubin (mg/dL), median (IQR)	245 (62)	0.5 (0.4–0.7)
Albumin (mg/dL), mean (SD)	235 (59)	42.8 (5.4)
AST (IU/L), median (IQR)	277 (70)	44 (31–69)
ALT (IU/L), median (IQR)	286 (72)	52.5 (33–88)
>ULN, *n* (%)		174 (60.8)
AP (IU/L), median (IQR)	244 (61)	83 (66.8–107)
gGT (IU/L), median (IQR)	283 (71)	48 (27–109)
>ULN, *n* (%)		140 (49.5%)
CRP, mg/dL, median (IQR)	227 (57)	0.2 (0.1–0.4)

Abbreviations: ALT, alanine aminotransferase; AP, alkaline phosphatase; APRI, AST to PLT ratio‐index; AST, aspartate aminotransferase; BL, baseline; CAP, continuous attenuation parameter; CRP, C‐reactive protein; DAA, direct acting agent; gGT, gammaglutamyl transferase; HCV, hepatitis C virus; HIV, human immunodeficiency virus; INR, international normalised ratio; IQR, interquartile range; LSM, liver stiffness measurement; n, number; SD, standard deviation; ULN, upper limit of normal; WBC, white‐cell blood count.

Of note, 6 (1.8%) individuals showed a chronic HBV‐coinfection, none of whom had detectable HBV‐DNA via PCR. HIV‐coinfection was more common (*n* = 51, 15.6%). Common comorbidities were significant alcohol intake (42.1%), cigarette smoking (51.1%), ongoing injecting drug use (45.0%), psychiatric disorders (21.9%), opioid agonist therapy (23.5%), and overweight or obese (45.7% and 13.7%, defined by a BMI of ≥25/30 kg/m^2^). According to non‐invasive assessment by continuous attenuation parameter (CAP) of ≥248 dB/m, 226 individuals (33.6%) had hepatic steatosis. Transient elastography revealed elevated LSM (i.e. ≥15 kPa) in 39 of 238 individuals (16.4%).

Using the APRI score, significant fibrosis could be ruled out in 204 of 275 individuals (74.2%), while 31 of 275 (11.3%) were at risk of significant fibrosis. According to the previously described cut‐offs of the FIB‐4 score, 62 of 275 (22.5%) individuals were at risk of significant fibrosis. Overall, 74 of 298 individuals (24.8%) met one of these NIT cut‐offs for significant fibrosis risk, at treatment initiation (Table 2).

### 3.4 Outcomes

Of the 397 individuals who were started on DAA treatment, 326 (82.1%) have achieved SVR (Table 3).

**TABLE 3:** Patient characteristics at the confirmation of sustained virological response (SVR).

	Available data, *n* (%)	Overall (*n* = 326)
*Patient characteristics*		
Sex, *n* (%)		
Female	326 (100)	204 (62.6)
Male		122 (37.4)
LSM (kPa), median (IQR)	116 (35.6)	5.9 (4.4–10.4)
LSM ≥ 10 kPa, *n* (%)		30 (9.2)
LSM ≥ 15 kPa, *n* (%)		17 (5.2)
LSM ≥ 25 kPa, *n* (%)		8 (2.5)
CAP (dB/m), median (IQR)		226 (192–271)
CAP ≥248 dB/m, *n* (%)		37 (11.3)
CAP >275 dB/m, *n* (%)		23 (7.1)
APRI score, median (IQR)		0.26 (0.18–0.37)
<0.5, *n* (%)	209 (64.1)	176 (54.0)
<1.0, *n* (%)		193 (59.2)
>1.5, *n* (%)		8 (2.5)
>2.0, *n* (%)		5 (1.5)
FIB‐4 score, median (IQR)		1.34 (0.84–1.96)
<1.3, *n* (%)	207 (63.5)	100 (30.7)
1.3–2.67, *n* (%)		80 (24.5)
>2.67, *n* (%)		27 (8.3)
Haemoglobin (g/dL), mean (SD)	212 (65.0)	13.9 (1.6)
Platelet count (G/L), median (IQR)	212 (65.0)	209 (168–255)
WBC (G/L), median (IQR)	212 (65.0)	6.7 (5.4–8.4)
INR, mean (SD)	193 (59.2)	1.1 (0.2)
Bilirubin (mg/dL), median (IQR)	212 (65.0)	0.4 (0.3–0.7)
Albumin (mg/dL), mean (SD)	209 (64.1)	44.0 (4.2)
AST (IU/L), median (IQR)	205 (62.9)	23 (18–29)
ALT (IU/L), median (IQR)	205 (62.9)	20 (14–27)
>ULN, *n* (%)		13 (4.0)
AP (IU/L), median (IQR)	212 (65.0)	75 (62–93)
gGT (IU/L), median (IQR)	212 (65.0)	21 (15–35)
> ULN, *n* (%)		28 (8.6)
CRP (mg/dL), median (IQR)	149 (58)	0.1 (0.1–0.4)

Abbreviations: ALT, alanine aminotransferase; AP, alkaline phosphatase; APRI, AST to PLT ratio‐index; AST, aspartate aminotransferase; CAP, continuous attenuation parameter; CRP, C‐reactive protein; gGT, gammaglutamyl transferase; HCV, hepatitis C virus; INR, international normalised ratio; IQR, interquartile range; LSM, liver stiffness measurement; *n*, number; SD, standard deviation; SVR, sustained virological response; ULN, upper limit of normal; WBC, white‐cell blood count.

**FIGURE 3:**
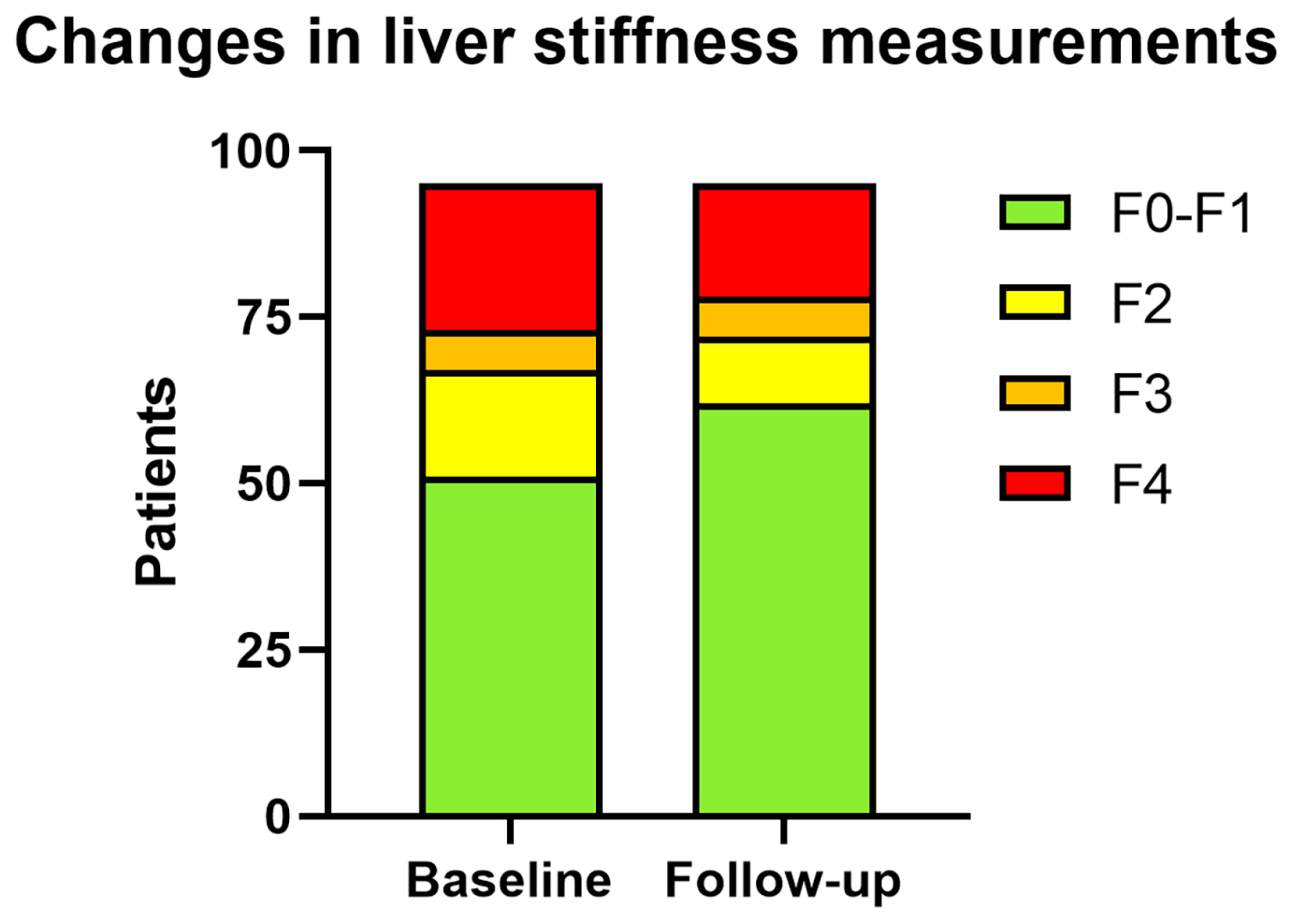
Comparative analysis of LSM pre‐ and post‐antiviral treatment. LSM by vibration‐controlled transient elastography in kPa among 95 patients with available LSM data. DAA, direct acting antiviral agents; LSM, liver stiffness measurement SVR, sustained virological response.

Elevated LSM values after successful treatment were rare at 30 of 116 available LSM readings. Additional information and parameters at the time of SVR are specified in Table 3. A comparison of paired LSM readings before and after treatment (*n* = 95) showed a statistically significant decrease in LSM values (baseline median LSM: 6.5 [IQR 5.4–9.9] vs. follow‐up median LSM: 6.1 [IQR 4.5–8.9]; Wilcoxon Rank test: *p* < 0.001) at SVR, as illustrated in Figure 3.

At the time of contact, 1975 (34.7%) individuals had passed away (Table 4). Of these, 667 (33.8%) were attributable to liver‐related and 287 (14.5%) were related to psychiatric comorbidity. The median age at death was 63 ± 17.9 years, with most deceased individuals being over 50 years old (*n* = 1395, 70.6%).

**TABLE 4:** Patient outcomes and mortality data for the overall cohort.

	Overall (*n* = 5695)
*Patient characteristics*	
Deaths, *n* (%)	1975 (34.7)
Liver‐related death, *n* (%)	667 (33.8)
Death related to psychiatric comorbidities, *n* (%)	287 (14.5)
Age at death, years, mean (SD)	63 (17.9)
≥50 years, *n* (%)	1395 (70.6)
Deaths during COVID period, *n* (%)	324 (16.4)
Deaths according to sex, *n* (%)	
Female	781 (36.6)
Male	1194 (33.5)

**FIGURE 4:**
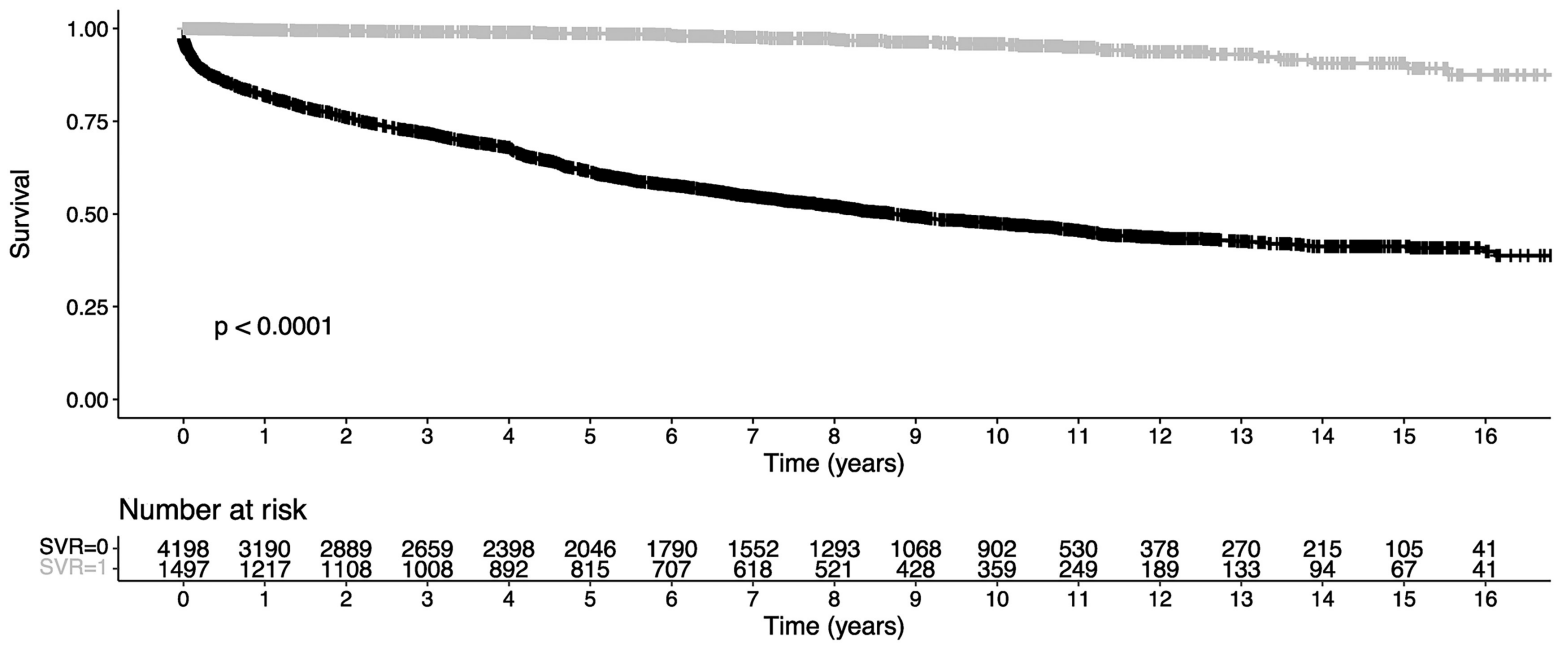
Comparison of Kaplan–Meier curves of individuals who achieved versus did not achieve sustained virological response (SVR).

Figure 4 compares the survival according to the SVR status. Individuals who did not achieve SVR had a median overall survival of 8.75 years (95%CI: 8.11–9.58) versus not reached in individuals who achieved SVR (*p* < 0.001).

The COVID‐19 pandemic (i.e. after 15 March 2020), which covered 22.6% of the study period, accounted for 16.4% of the deaths. Psychiatric morbidity‐related deaths tended to be more common during the COVID pandemic, compared to the pre‐pandemic period (19.1% vs. 14.9%; *p* = 0.059). Contrarily, liver‐related deaths were less common during the COVID pandemic (23.1% vs. 38.7%; *p* < 0.001). Individuals with psychiatric comorbidities died significantly younger (on average 20 years younger) compared to individuals without (diagnosed) psychiatric comorbidities (mean age: 46.1 vs. 66.2 years; *p* < 0.001). Additionally, male individuals who died were, on average, 10 years younger than their female counterparts (mean age at death: 59.1 vs. 69.3 years; *p* < 0.001; Table 4).

## 4 DISCUSSION

Despite highly effective antiviral treatment options for hepatitis C, achieving the WHO's goal of eliminating hepatitis C by 2030 remains challenging as re‐connecting with individuals lost to care represents a significant hurdle. Previous studies indicate that despite the availability of DAAs, only one third of individuals diagnosed with HCV infection receive antiviral treatment, with the majority of them being lost to follow‐up.^1^ To address this issue, we launched the ELIMINATE (ELIMINation AusTria East) project, a first of its kind macro‐elimination program in Austria. This initiative was uniquely based on systematic laboratory record‐based identification of people with readily available ‘last‐positive’ HCV‐RNA PCR results followed by a comprehensive re‐call strategy in 10 tertiary care centres in Eastern Austria.^31^ In contrast to previous Austrian micro‐elimination projects targeting populations who are considered specifically vulnerable to HCV infection, the ELIMINATE project is agnostic of risk behaviour as it is just based on HCV viraemia and thus, potentially reaching a broader spectrum of individuals affected by HCV.

The ELIMINATE project allowed us to identify 5695 persons with previous HCV‐RNA viraemia, enabling us to contact a significant number of people living with HCV. Despite challenges such as invalid contact information and deceased individuals, we found that an encouraging number of 1171 individuals had already achieved viral clearance. Our two‐step approach of contacting 3005 individuals, first, by phone and, if not reached, subsequently by mailed letter, resulted in a total of 617 people living with HCV and partly unaware of their infection responding to our re‐call. Of these, a remarkable 417 scheduled visits and 397 (64.3%) HCV viraemic individuals started DAA therapy, demonstrating an outstanding rate of linkage to care as two‐thirds of the contacted individuals received DAA therapy. This achievement stands out when compared to similar studies: one Taiwanese ‘callback’ study^28^ based on HCV‐antibodies contacted 11 934 of 31 275 identified HCV‐antibody‐positive individuals, with only 323 (2.7% of contacted individuals) receiving DAA‐treatment. A Dutch study in Alkmaar,^38^ using public health service data, identified 499 potential HCV‐RNA‐positive individuals, with only three (12.5%) of 24 eligible individuals starting antiviral therapy. Another Dutch study in Utrecht^39^ analysed hospital and laboratory data regarding anti‐HCV‐positive and/or HCV RNA‐positive individuals, inviting 269 re‐discovered individuals, with 22 (8.1%) commencing DAA therapy. Similarly, a Brazilian study^40^ analysed the patient registries of three large outpatient clinics and successfully contacted 1818 HCV‐RNA PCR‐positive individuals either by phone or by mailed letter, with 201 (11.5%) receiving antiviral treatment. Our project was based on already available PCR data and thus, circumvented potential extra steps associated with an antibody‐based re‐call system, where many non‐viraemic individuals are identified as well. These comparisons highlight the effectiveness of the ELIMINATE project in linking individuals to care and initiating treatment. Although we could not show data on cost‐efficacy in our study, implementing this macro‐elimination study design into clinical routine in participating centres facilitated HCV cure (SVR) in a considerable proportion of individuals. With the high number of deaths prior to re‐call, this study also underlines the need to implement such elimination strategies into clinical routine to reach the WHO goals.

Our findings highlight the vulnerability of the HCV patient population, as evidenced by the fact that at the time of data acquisition, 1931 (34%) of the potentially eligible HCV individuals had already passed away. Common health hazards and comorbidities prevalent in our overall population included pathological alcohol use, cigarette smoking, ongoing injecting drug use and psychiatric disorders. Our study results emphasise that a substantial proportion of individuals have only been linked to care for the specific disease that was individually perceived as their main priority, for example, opioid substitution or HIV coinfection, but were widely not aware of their chronic HCV infection and/or of the availability of highly effective DAA therapies.

Notably, only one third of the reported deaths in our HCV population were documented to be liver‐related while death related to psychiatric comorbidities emerged as the second most common cause of mortality. This data points to the multifaceted health challenges faced by individuals living with HCV, underscoring the need for comprehensive healthcare approaches that address both physical and mental health needs.

Furthermore, we observed that among individuals already aware of their chronic HCV infection, common barriers to receiving DAA therapy or being referred to a treatment centre included lack of awareness about the availability of DAA therapy, possibly compounded by apprehension stemming from previous experiences with interferon treatments. Additionally, traditional reimbursement policies in Austria, which limited DAAs to individuals with fibrosis stage ≥F2, acted as a deterrent. Other factors such as advanced age, ongoing alcohol consumption or injecting drug use also played a role in hindering access to treatment.

Importantly, treating HCV infection in individuals with ongoing risk behaviour is both safe and effective and limits further transmission of HCV—a concept known as ‘treatment as prevention’.^41^ Moreover, while individuals with alcohol use disorder are less likely to receive DAA treatment, it has been shown that alcohol use does not significantly impact the safety or efficacy of these drugs.^42^ Rather, concomitant alcohol use is a main driver for liver disease progression in individuals with chronic hepatitis C; hence, antiviral treatment for HCV should not be delayed.^43^ Nevertheless, adherence to DAAs may be impaired in individuals with psychiatric comorbidities including substance use disorders, therefore, it may be necessary to adapt treatment approaches and provide individual supporting measures to attend the specific needs of these vulnerable populations.^44^ In this regard, healthcare provider education and creating awareness have been shown to be crucial steps to improve linkage to care.^22^

Our macro‐elimination program is the first of its kind in Austria and stands as an excellent example of successful cooperation between multiple hospitals. While this effort led to the identification and successful treatment of a significant number of individuals with HCV, the success of this program largely hinged on the dedication and perseverance of healthcare professionals at each participating centre. The impact of national elimination programs is evident in global examples such as Egypt. Previously ranked fifth worldwide in HCV prevalence, Egypt treated 3.5 million citizens from 2015 to 2019 following its national elimination plan, subsequently dropping to rank 17 on global rankings by 2020.^45^ We believe that with potent tools like DAAs available, powerful national elimination plans tracing the progress and implementing comprehensive interventions encompassing all healthcare facilities are needed.

Another obstacle we face in Austria and other European countries are restrictions to DAA prescription due to insurance coverage.^26^ Prescription of DAA treatment is often limited to certain centres and mostly to the outpatient setting, thus impeding the streamlined initiation of HCV treatment and potentially breaking an established linkage to care. To enhance linkage to care, the liver outpatient clinic of the Vienna General Hospital has established a dedicated HCV hotline. Operated by physicians and nurses, this hotline provides a direct communication channel for people living with HCV or those with suspected viral hepatitis. It allows individuals to directly contact healthcare professionals and schedule appointments for diagnostic evaluations and/or the initiation of DAA therapy.^32^, ^33^ This physician‐ and nurse‐operated HCV hotline was an essential component of our ELIMINATE project. Cost efficiency does not justify a 24‐h hotline covered by medical doctors. However, phone calls by medical doctors were performed during working hours. On top, prevention of complications of ongoing HCV infection must be the ultimate goal, decreasing costs inherently.

Our study has some limitations. Prior SVR status was mostly confirmed by negative PCR data, but was self‐reported in some rare cases, and hence, some individuals may still have HCV infection. In individuals with self‐reported SVR, several details regarding the treatment were assessed to ensure the validity of the self‐reported outcome: (i) if the treatment was ‘with injections/interferon’, (ii) the name of the DAA used, (iii) the number of tablets per day and/or the duration of the treatment, (iv) the dedicated centre prescribing DAA therapy, (v) the year of the treatment. If the individual was able to provide coherent information regarding their treatment the self‐reported SVR was accepted. Individuals with incoherent responses or in any case of uncertainty of treatment status, visits were offered for check‐up and blood work at the respective centre. In order to ensure uniform and standardised procedures across the centres, regular virtual and presential meetings with members of all participating centres were held.

In Europe, combined elimination efforts resulted in an enormous increase in the number of HCV treatments over the last decade alongside a decrease in HCV incidence.^45^ Concordantly, the European Disease Centre reported a decline in hepatitis C notification rate, dropping from 8.8 per 100 000 individuals in 2016 to 3.9 in 2020.^12^ Despite this positive trend, the numbers from our Austrian elimination program showed that a substantial number of potential HCV viraemic individuals remain untreated and concealed within the general population, underscoring the ongoing need for targeted identification and treatment efforts.

Our project showed that systematic identification of individuals with positive PCR for HCV‐RNA from pre‐existing laboratory data, constitutes an effective and reproducible approach for HCV macro‐elimination. Through the ELIMINATE program, we were able to successfully identify and provide care to individuals living with hepatitis C irrespective of risk behaviour. This successful, systematic, PCR‐based macro‐elimination strategy appears broadly applicable to other countries, offering a powerful tool on the road towards the WHO's goal of eliminating viral hepatitis by 2030.

## AUTHOR CONTRIBUTIONS

Concept of the study (D.B., C.S., T.R., M.G.), data collection (all authors), statistical analyses (L.B. and M.S.), drafting of the manuscript (L.B., M.S.) and revision for important intellectual content and approval of the final manuscript (all authors).

## FUNDING INFORMATION

This study was supported by a restricted research grant (ELIMINATE: Eliminiation of Austria East) from Gilead Sciences awarded to Thomas Reiberger.

## CONFLICT OF INTEREST STATEMENT

The authors have nothing to disclose regarding the work under consideration for publication. L.D., M.J., L.H., L.W., N.P., B.H., A.S., R.S., A.V.‐G., W.H., M.W., K.K., S.J.‐S., S.T., W.K., C.M., L.A., L.Bu., G.W., F.R., H.L., J.H., C.W., J.W.‐W., P.W., M.N., D.S., J.R. and S.R. have nothing to disclose outside of the submitted work. L.B. received speaking honoraria from Chiesi and Gilead. M.S. received travel support from MSD, Sandoz, BMS, AbbVie and Gilead; and speaking honoraria from BMS. D.C. served as a speaker and/or advisory board member for Gilead, ViiV Healthcare and MSD and received travel support from MSD, ViiV Healthcare and Gilead. M.M. served as a speaker and/or consultant and/or advisory board member for AbbVie, Collective Acumen, Echosens, Gilead, Takeda and W. L. Gore & Associates and received travel support from AbbVie and Gilead. M.G. received grant support from AbbVie, Gilead and MSD; speaking honoraria from AbbVie, Gilead, Janssen, Roche, Intercept and MSD; consulting/advisory board fees from AbbVie, Gilead, Janssen, Roche, Intercept, Norgine, AstraZeneca, Falk, Shionogi and MSD; and travel support from AbbVie and Gilead. A.M. received grant support from AbbVie and Gilead; speaking honoraria from AbbVie, Gilead, Janssen, Roche, Intercept, and MSD; consulting/advisory board fees from AbbVie, Gilead, Janssen, Roche, Intercept, Norgine and MSD; and travel support from AbbVie, Gilead and Roche. T.R. served as a speaker and/or consultant and/or advisory board member speaking honoraria from AbbVie, Bayer, Boehringer‐Ingelheim, Gilead, Intercept, MSD, Roche, Siemens and W. L. Gore & Associates and received travel support from AbbVie, Boehringer‐Ingelheim, Gilead and Roche as well as grants/research support from AbbVie, Boehringer‐Ingelheim, Gilead, Intercept, MSD, Myr Pharmaceuticals, Philips Healthcare, Pliant, Siemens and W. L. Gore & Associates. D.B. received travel support from Gilead and AbbVie; speaking honoraria from AbbVie and Siemens and grant support from Gilead and AbbVie. C.S. received travel support from Gilead, AbbVie, Galápagos and Gebro; speaking honoraria from AbbVie and Gilead; and payments for consulting from Gilead.

## Data Availability

The data that support the findings of this study are available from the corresponding author upon reasonable request.

## References

[1] EASL recommendations on treatment of hepatitis C: final update of the series. J Hepatol. 2020;73(5):1170‐1218. 32956768 10.1016/j.jhep.2020.08.018

[2] Westbrook RH , Dusheiko G . Natural history of hepatitis C. J Hepatol. 2014;61(1 Suppl):S58‐S68. 25443346 10.1016/j.jhep.2014.07.012

[3] Martinello M , Solomon SS , Terrault NA , Dore GJ . Hepatitis C. Lancet. 2023;402(10407):1085‐1096. 37741678 10.1016/S0140-6736(23)01320-X

[4] Manns MP , Wedemeyer H , Cornberg M . Treating viral hepatitis C: efficacy, side effects, and complications. Gut. 2006;55(9):1350‐1359. 16905701 10.1136/gut.2005.076646 PMC1860034

[5] Lawitz E , Mangia A , Wyles D , et al. Sofosbuvir for previously untreated chronic hepatitis C infection. N Engl J Med. 2013;368(20):1878‐1887. 23607594 10.1056/NEJMoa1214853

[6] Falade‐Nwulia O , Suarez‐Cuervo C , Nelson DR , Fried MW , Segal JB , Sulkowski MS . Oral direct‐acting agent therapy for hepatitis C virus infection: a systematic review. Ann Intern Med. 2017;166(9):637‐648. 28319996 10.7326/M16-2575 PMC5486987

[7] World Health Organization . Global health sector strategy on viral hepatitis 2016–2021 towards ending viral hepatitis 2016.

[8] Heffernan A , Cooke GS , Nayagam S , Thursz M , Hallett TB . Scaling up prevention and treatment towards the elimination of hepatitis C: a global mathematical model. Lancet. 2019;393(10178):1319‐1329. 30704789 10.1016/S0140-6736(18)32277-3 PMC6484702

[9] Viner K , Kuncio D , Newbern EC , Johnson CC . The continuum of hepatitis C testing and care. Hepatology. 2015;61(3):783‐789. 25348499 10.1002/hep.27584

[10] Safreed‐Harmon K , Blach S , Aleman S , et al. The consensus hepatitis C Cascade of care: standardized reporting to monitor progress toward elimination. Clin Infect Dis. 2019;69(12):2218‐2227. 31352481 10.1093/cid/ciz714

[11] Austrian Federal Ministry of Social Affairs Health Care and Consumer Protection . HIV/AIDS, Hepatitis B and C in Austria. 2019.

[12] European Centre for Disease Prevention and Control . Hepatitis C Annual Epidemiological Report for 2020. ECDC. 2022.

[13] Schmidbauer C , Schubert R , Schütz A , et al. Directly observed therapy for HCV with glecaprevir/pibrentasvir alongside opioid substitution in people who inject drugs‐first real world data from Austria. PLoS One. 2020;15(3):e0229239. 32155165 10.1371/journal.pone.0229239 PMC7064180

[14] Schwarz M , Schwarz C , Schütz A , et al. Combining treatment for chronic hepatitis C with opioid agonist therapy is an effective microelimination strategy for people who inject drugs with high risk of non‐adherence to direct‐acting antiviral therapy. J Virus Erad. 2023;9(1):100319. 36970063 10.1016/j.jve.2023.100319 PMC10036924

[15] Schmidbauer C , Schwarz M , Schütz A , et al. Directly observed therapy at opioid substitution facilities using sofosbuvir/velpatasvir results in excellent SVR12 rates in PWIDs at high risk for non‐adherence to DAA therapy. PLoS One. 2021;16(6):e0252274. 34086708 10.1371/journal.pone.0252274 PMC8177501

[16] Jachs M , Binter T , Chromy D , et al. Outcomes of an HCV elimination program targeting the Viennese MSM population. Wien Klin Wochenschr. 2021;133(13–14):635‐640. 34181068 10.1007/s00508-021-01898-9 PMC8237255

[17] Schwarz M , Gremmel S , Wurz M , et al. “Let's end hepatitis C in Vienna”—the first HCV elimination program targeting homeless and people without medical insurance in Vienna. Z Gastroenterol. 2020;58(5):P61.

[18] Silbernagl M , Slamanig R , Fischer G , Brandt L . Hepatitis C infection and psychiatric burden in two imprisoned cohorts: young offenders and opioid‐maintained prisoners. Health Policy. 2018;122(12):1392‐1402. 30392782 10.1016/j.healthpol.2018.10.005

[19] Lazarus JV , Wiktor S , Colombo M , Thursz M , EASL International Liver Foundation . Micro‐elimination—a path to global elimination of hepatitis C. J Hepatol. 2017;67(4):665‐666. 28760329 10.1016/j.jhep.2017.06.033

[20] Thomas DL , Longo DL . Global elimination of chronic hepatitis. N Engl J Med. 2019;380(21):2041‐2050. 31116920 10.1056/NEJMra1810477

[21] Mangia A , Cotugno R , Cocomazzi G , Squillante MM , Piazzolla V . Hepatitis C virus micro‐elimination: where do we stand? World J Gastroenterol. 2021;27(16):1728‐1737. 33967553 10.3748/wjg.v27.i16.1728 PMC8072193

[22] Cunningham EB , Wheeler A , Hajarizadeh B , et al. Interventions to enhance testing, linkage to care, and treatment initiation for hepatitis C virus infection: a systematic review and meta‐analysis. Lancet Gastroenterol Hepatol. 2022;7(5):426‐445. 35303490 10.1016/S2468-1253(21)00471-4

[23] Huang CF , Chen GJ , Hung CC , Yu ML . HCV microelimination for high‐risk special populations. J Infect Dis. 2023;228(Suppl 3):S168‐S179. 37703340 10.1093/infdis/jiac446

[24] Taha G , Ezra L , Abu‐Freha N . Hepatitis C elimination: opportunities and challenges in 2023. Viruses. 2023;15(7):1413. 37515101 10.3390/v15071413 PMC10386528

[25] The European Union HCV Collaborators . Hepatitis C virus prevalence and level of intervention required to achieve the WHO targets for elimination in the European Union by 2030: a modelling study. Lancet Gastroenterol Hepatol. 2017;2(5):325‐336. 28397696 10.1016/S2468-1253(17)30045-6

[26] Aleman S , Soderholm J , Busch K , et al. Frequent loss to follow‐up after diagnosis of hepatitis C virus infection: a barrier towards the elimination of hepatitis C virus. Liver Int. 2020;40(8):1832‐1840. 32294288 10.1111/liv.14469

[27] Stasi C , Silvestri C , Voller F . Update on hepatitis C epidemiology: unaware and untreated infected population could Be the key to elimination. SN Compr Clin Med. 2020;2(12):2808‐2815. 33103061 10.1007/s42399-020-00588-3 PMC7568689

[28] Chen CJ , Huang YH , Hsu CW , et al. Hepatitis C micro‐elimination through the retrieval strategy of patients lost to follow‐up. BMC Gastroenterol. 2023;23(1):40. 36782112 10.1186/s12876-023-02665-y PMC9926801

[29] Lopes H , Baptista‐Leite R , Franco D , Serra MA , Escudero A , Martín‐Moreno JM . Let's end HepC: modelling public health epidemiological policies applied to hepatitis C in Spain. Front Public Health. 2021;9:735572. 35071151 10.3389/fpubh.2021.735572 PMC8777247

[30] Omran D , Alboraie M , Zayed RA , et al. Towards hepatitis C virus elimination: Egyptian experience, achievements and limitations. World J Gastroenterol. 2018;24(38):4330‐4340. 30344418 10.3748/wjg.v24.i38.4330 PMC6189850

[31] Schwarz C , Bauer D , Dorn L , et al. ELIMINATE: a PCR record‐based macroelimination project for systematic recall of HCV‐RNA‐positive persons in Austria. Wien Klin Wochenschr. 2023;136:278‐288. 37773541 10.1007/s00508-023-02275-4 PMC11078856

[32] Hartl L , Jachs M , Bauer D , et al. HCV hotline facilitates hepatitis C elimination during the COVID‐19 pandemic. J Viral Hepat. 2022;29(12):1062‐1072. 36062398 10.1111/jvh.13746 PMC9825935

[33] Steininger L , Chromy D , Bauer D , et al. Direct patient‐physician communication via a hepatitis C hotline facilitates treatment initiation in patients with poor adherence. Wien Klin Wochenschr. 2021;133(9–10):452‐460. 33351152 10.1007/s00508-020-01790-y PMC8116284

[34] de Franchis R , Bosch J , Garcia‐Tsao G , et al. Baveno VII—renewing consensus in portal hypertension. J Hepatol. 2022;76(4):959‐974. 35120736 10.1016/j.jhep.2021.12.022 PMC11090185

[35] Castera L , Forns X , Alberti A . Non‐invasive evaluation of liver fibrosis using transient elastography. J Hepatol. 2008;48(5):835‐847. 18334275 10.1016/j.jhep.2008.02.008

[36] Wai CT , Greenson JK , Fontana RJ , et al. A simple noninvasive index can predict both significant fibrosis and cirrhosis in patients with chronic hepatitis C. Hepatology. 2003;38(2):518‐526. 12883497 10.1053/jhep.2003.50346

[37] Shah AG , Lydecker A , Murray K , et al. Comparison of noninvasive markers of fibrosis in patients with nonalcoholic fatty liver disease. Clin Gastroenterol Hepatol. 2009;7(10):1104‐1112. 19523535 10.1016/j.cgh.2009.05.033 PMC3079239

[38] Beekmans N , Klemt‐Kropp M . Re‐evaluation of chronic hepatitis B and hepatitis C patients lost to follow‐up: results of the Northern Holland hepatitis retrieval project. Hepatol Med Policy. 2018;3(1):5. 30288328 10.1186/s41124-018-0032-9 PMC5918904

[39] Kracht PAM , Arends JE , van Erpecum KJ , et al. REtrieval and cure of chronic hepatitis C (REACH): results of micro‐elimination in the Utrecht province. Liver Int. 2019;39(3):455‐462. 30204289 10.1111/liv.13959

[40] Ferraz MLG , de Andrade A , Pereira GHS , et al. Retrieval of HCV patients lost to follow‐up as a strategy for hepatitis C microelimination: results of a Brazilian multicentre study. BMC Infect Dis. 2023;23(1):468. 37442976 10.1186/s12879-023-08169-0 PMC10339629

[41] Hajarizadeh B , Grebely J , Martinello M , Matthews GV , Lloyd AR , Dore GJ . Hepatitis C treatment as prevention: evidence, feasibility, and challenges. Lancet Gastroenterol Hepatol. 2016;1(4):317‐327. 28404202 10.1016/S2468-1253(16)30075-9

[42] Cartwright EJ , Pierret C , Minassian C , et al. Alcohol use and sustained virologic response to hepatitis C virus direct‐acting antiviral therapy. JAMA Netw Open. 2023;6(9):e2335715. 37751206 10.1001/jamanetworkopen.2023.35715 PMC10523171

[43] Alavi M , Janjua NZ , Chong M , et al. The contribution of alcohol use disorder to decompensated cirrhosis among people with hepatitis C: an international study. J Hepatol. 2018;68(3):393‐401. 29107152 10.1016/j.jhep.2017.10.019

[44] Schwarz C , Schubert R , Schwarz M , et al. CHIME—a tailored HCV microelimination project in Viennese people who inject drugs at drug centralized substitution centers. J Virus Erad. 2023;9(3):100338. 37663576 10.1016/j.jve.2023.100338 PMC10474458

[45] Polaris Observatory HCVC . Global change in hepatitis C virus prevalence and cascade of care between 2015 and 2020: a modelling study. Lancet Gastroenterol Hepatol. 2022;7(5):396‐415. 35180382 10.1016/S2468-1253(21)00472-6

